# SMU Open-Source Platform for Synchronized Measurements

**DOI:** 10.3390/s22145074

**Published:** 2022-07-06

**Authors:** Carlo Guarnieri Calò Carducci, Marco Pau, Cesar Cazal, Ferdinanda Ponci, Antonello Monti

**Affiliations:** 1 Institute for Automation of Complex Power Systems, RWTH Aachen University, 52074 Aachen, Germany; cesar.cazal@eonerc.rwth-aachen.de (C.C.); fponci@eonerc.rwth-aachen.de (F.P.); amonti@eonerc.rwth-aachen.de (A.M.); 2 Fraunhofer Institute for Energy Economics and Energy System Technology, 34117 Kassel, Germany; marco.pau@iee.fraunhofer.de; 3 Fraunhofer Institute for Applied Information Technology, 52074 Aachen, Germany

**Keywords:** single-board computer, data acquisition systems, distributed monitoring, low-cost measurement units, synchronized measurements, phasor measurement unit

## Abstract

The ramping trend of cheap and performant single board computers (SBC) is growingly offering unprecedented opportunities in various domains, taking advantage of the widespread support and flexibility offered by an operating system (OS) environment. Unfortunately, data acquisition systems implemented in an OS environment are traditionally considered not to be suitable for reliable industrial applications. Such a position is supported by the lack of hardware interrupt handling and deterministic control of timed operations. In this study, the authors fill this gap by proposing an innovative and versatile SBC-based open-source platform for CPU-independent data acquisition. The synchronized measurement unit (SMU) is a high-accuracy device able to perform multichannel simultaneous sampling up to 200 kS/s with sub-microsecond synchronization precision to a GPS time reference. It exhibits very low offset and gain errors, with a minimum bandwidth beyond 20 kHz, SNR levels above 90 dB and THD as low as −110 dB. These features make the SMU particularly attractive for the power system domain, where synchronized measurements are increasingly required for the geographically distributed monitoring of grid operating conditions and power quality phenomena. We present the characterization of the SMU in terms of measurement and time synchronization accuracy, proving that this device, while low-cost, guarantees performance compliant with the requirements for synchrophasor-based applications in power systems.

## 1. Introduction

With the spread of distributed energy resources connected to the distribution level of the electric system, operating the power grid is becoming an increasingly complex task that requires automated control down to the low voltage grid [1]. To this purpose, measurements are essential to unlock system awareness. Nowadays, however, distribution systems are typically still poorly instrumented grids, with few devices often installed only in the primary substations [2]. This calls for a significant upgrade of the power system measurement infrastructure and for the deployment of devices able to fulfil the new requirements emerging in the power system scenario.

The deployment of measurements, in particular at the distribution grid level, has indeed very specific requirements. The first one is associated with the cost of the measurement units. Distribution systems have a very large number of nodes and, therefore, a huge number of devices would be required to achieve full measurement coverage. Since this is economically unfeasible, optimal meter placement strategies are commonly adopted to find the best tradeoff among the number, cost, and accuracy of the devices to be installed [3,4,5,6]. Reducing the costs of the measurement units clearly helps to reduce the overall costs or to increase the number of installations with a given budget. For this reason, in the last few years, several works have focused on the development of low-cost measurement units [7].

The OpenPMU project [8] provided one of the first contributions, by releasing open-source specifications and software for the design of low-cost phasor measurement units (PMUs). The work in [9] presented instead a PMU prototype based on field programmable gate array (FPGA). This solution, while cheaper than commercial solutions, aimed to achieve high performance and for this reason was still based on relatively expensive hardware. Cheaper PMUs based on single-board computers (SBC) were proposed in [10], using a Raspberry Pi, in [11], using a Beagle Bone Black, and in [12], adopting an ARM microcontroller. Recently, an improved PMU design based on Raspberry Pi has been described in [13]. Low-cost smart meters and power quality analyzers (PQAs) have been also proposed, for example in [14] and [15], respectively, while Orlando et al. [16] presented a low-cost three-phase IoT node that combines metering and processing. The above works proved the feasibility of leveraging the increasing performance of SBCs to develop measurement devices based on general purpose and relatively low-cost hardware.

Close to the low-cost requirement, another important aspect concerns the types of measurements to be collected from the grid. In this regard, it is worth highlighting the fact that different applications may have quite different measurement requirements [17]. As an example, digital smart meters recently installed in many countries are suitable for energy billing, but they are not appropriate for real-time monitoring with fine-grained time resolution. PMUs provide synchronized measurements with high reporting rate that are useful for many real-time management and automation functions, but they do not give an insight on several power quality issues; for this application PQAs would be rather needed. More recently, synchronized waveform measurement units (WMUs) have been proposed for advanced applications, such as dynamic monitoring, transient event detection and fault management [18]. Raw waveform samples may be essential for the aforementioned applications [19], but in general are not needed for many of the quasi-static control functions. Power systems are also increasingly integrating DC technologies. This leads to the need for new measurements in the DC system to support novel control applications in the hybrid AC/DC grid (see for example [20]). In this scenario, an emerging requirement is thus to have flexible (software-defined) measurement devices capable of running different measurement algorithms (depending on the upper-level applications envisioned by the grid operator), which are easy to reconfigure (if and when needed) and that can be updated on-the-fly with new algorithms and software patches or releases.

In this context, this paper presents the synchronized measurement unit (SMU), an innovative SBC-based data acquisition platform that combines the above requirements of high performance, low cost, and flexibility. The measurement device builds upon a Raspberry Pi as main hardware component, providing an extremely powerful and versatile environment at a very limited cost. In the SMU, an elegant and efficient use of its resources allows to perform high-speed simultaneous multi-channel signal acquisition with no burden for the operating system, leaving more than 98% of the computational resources available for the specific application. The software, released open source together with the hardware design, is built in a modular way, decoupling the low-level functionalities needed for handling the data acquisition from the other libraries responsible for the measurement processing and for the data communication. The modular deployment of the software in the user space allows to easily adapt the device for running different measurement algorithms or adopting different communication protocols. In this way, the measurement unit can be flexibly configured to work as a WMU, PMU, or PQA, depending on the specific needs for the upper-level applications. Furthermore, the configuration can be changed at runtime and minimal software upgrades can potentially enable multiple data processing algorithms to run in parallel.

Overall, this paper brings the following novel contributions:

- It presents the optimized design of a generic Synchronized Measurement Unit, which relies on a Raspberry Pi 3 as main hardware component to ensure very low costs with industrial grade performance.
- It describes the hardware and software architecture designed to enable a plug-in based modular deployment of different functionalities, offering the possibility to flexibly swap different measurement algorithms and communication protocols.
- It shows the software suite implemented for the automated testing of the measurement device, together with related tests performed to characterize its accuracy and overall performance.

The following of this paper is organized as follows. Section 2 presents the general architecture of the SMU. Section 2.1 focuses on the hardware components, whereas Section 2.2 and Section 2.3 provide details of the SMU software layer and test environment, respectively. Section 3 describes the experimental setup adopted for the SMU characterization and the tests carried out to prove its capability to perform synchronized measurements in line with related power systems standards. Finally, Section 4 summarizes the main features of the SMU and provides the final remarks for this work.

## 2. Materials and Methods

**Figure 1:**
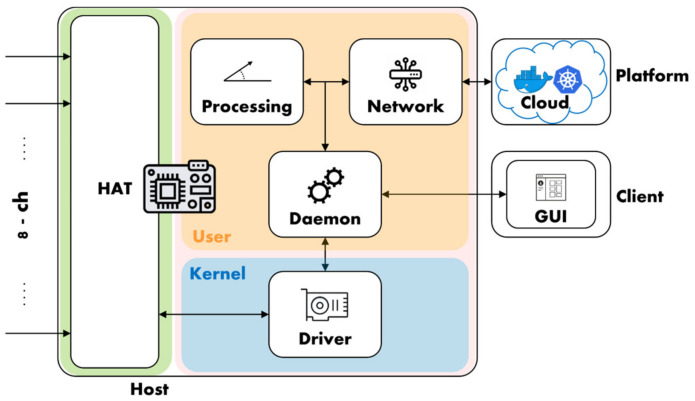
SMU architecture layers: hardware (green) and software (pink). The software layer is divided in kernel- (blue) and user-space (orange).

The SMU is a complete open-source data acquisition platform designed around the Raspberry Pi 3 single-board computer, in the following referred to as RPi3 for the sake of brevity. The presented platform encompasses several layers (Figure 1) hardware and software. Each of these layers is in turn composed of multiple sub-layers: the hardware includes the expansion board, or hardware-attached-on-top (HAT), and its device housing; the software spans from the driver which runs at low level in the kernel-space, to the daemon and its plugins that run at a higher level in the user-space. The platform also includes a complete MATLAB based software suite for automated testing.

For improved clarity of the presented architecture, layers and sub-layers will be described in the following in separate sections.

### 2.1. Hardware

The hardware layer is composed of an electronic and a mechanical sub-layer. The former (smuHAT), includes the electronic front-end block for signal acquisition, the time reference generation block, and the self-calibration block. The latter (smuBOX), includes the design of a plastic case for installation of the SMU on DIN rails.

#### 2.1.1. smuHAT

**Figure 2:**
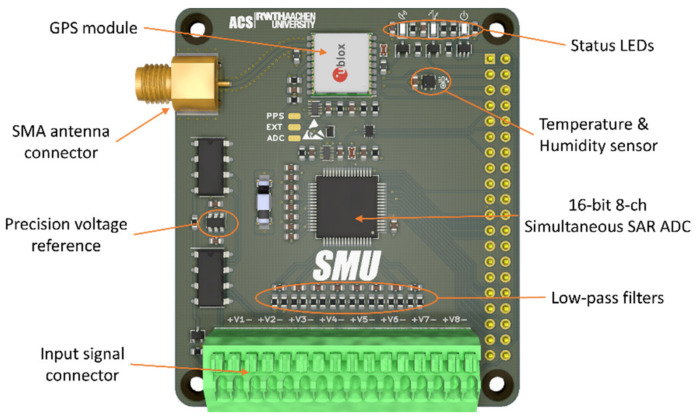
SMU hardware-attached-on-top module for Raspberry Pi 3 and main board components.

The SMU hardware-on-top (Figure 2) is an electronic board [21] designed to be mounted on the 40-pin header connector of a RPi3. The analog front-end (AFE) is based on the Texas Instruments ADS8588s module, a 16 bit 8 channels simultaneous sampling successive approximation register (SAR) analog-to-digital converter (ADC) module able to reach sample rates up to 200 kS/s. The integrated circuit (IC) already embeds a complete AFE for each channel, including a programmable gain amplifier with high impedance input (1 MΩ), input clamp and 3-rd order low-pass (LP) filter. However, an external array of differential LP filters can be configured upon need directly onboard to further reduce the ADC bandwidth, or to provide impedance matching with a specific sensor. The device supports multiple digital interfaces, including serial, parallel and parallel byte modes, the latter being adopted in the SMU design to achieve the maximum available sample rate, while occupying only 8 digital data pins.

The data transfer from the ADC, described more in detail later in Section 2.2.1, is autonomously managed by the secondary memory interface (SMI) block. This block is part of the hardware architecture of the Broadcom BCM2837 microprocessor, the CPU at the base of the RPi3 single-board computer. This transfer is paced by the ADC sampling base, also generated by the same CPU using the internal pulse-width modulator (PWM) block.

To achieve the desired synchronization of the time base with a precise reference, a GPS module u-blox MAX M8C is used to derive the pulse-per-second (PPS) time reference signal. Differently from traditional closed-loop hardware-based synchronization mechanisms such as phase-locked loops (PLL), the SMU uses the PPS signal to periodically trigger the sampling base generation in the form of a finite train of pulses. This results in a one-shot synchronization mechanism that repeats every second resetting those drift errors eventually cumulated in the previous period.

Both data transfer and time synchronization require extremely low latency interrupt detection, which is usually not possible to achieve in an operating system context. However, the SMI interface provides an elegant solution to overcome the lack of support of GPIO hardware interrupts. In fact, a data request (DREQ) pin can be configured to pace the data transfer, and when this mechanism is combined with a direct memory access (DMA) approach, it is possible to achieve sub-microsecond latencies in both data transfer and time synchronization. However, since the SMI data request interrupt is level-sensitive, the design features two monostable multivibrators 74LVC1G123 by Nexperia with Schmitt trigger inputs, which convert the edge- in level-sensitive triggers with a proper duration. An OR digital port is then used to combine the two triggers in one digital signal used to pace the DMA. Additionally, a third digital input allows to provide an external trigger source if needed.

The on-board system calibration block is based on the environmental sensor SHTC3 by Sensirion and on the REF3440T by Texas Instruments, a high precision voltage reference with low-drift (2.5 ppm/°C) and high-accuracy (0.05%). The sensor provides temperature and humidity readings useful to implement model-based compensation methods, for example accounting for the crystal oscillator (CXO) drift with temperature [22]. Similarly, the voltage reference can be used to evaluate the gain drift of the AFE with temperature, but also to evaluate the signal attenuation introduced by the custom impedance matching LP filters. The comparison is limited to the first channel though, therefore the compensation can be extended to other channels only under the assumption that observed variations affect similarly all channels, or, in other words, that the errors due to the inter-channel manufacturing tolerances are smaller than those due to external components.

#### 2.1.2. smuBOX

As the SMU is intended to be used in an industrial environment, a dedicated plastic enclosure with DIN-rail clamps was developed. Its compact design (9 cm × 6 cm × 2.6 cm) permits the installation in standard electrical cabinets even with little available space.

**Figure 3:**
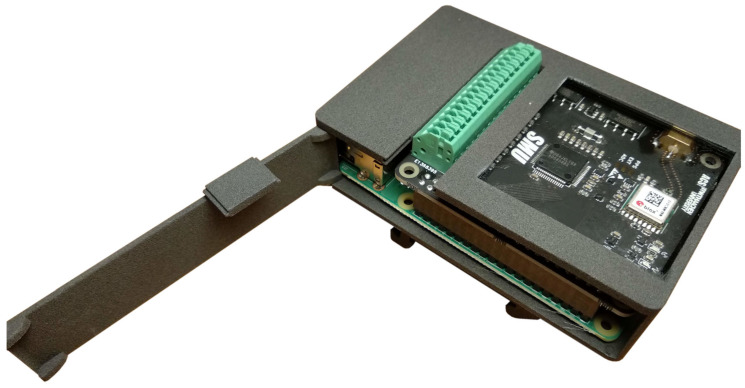
View of the 3D printed SMU enclosure; the side opening allows to easily slide inside/outside the Raspberry Pi with the smuHAT installed on top.

Design files are readily available [23] in STEP format to be directly submitted for 3D printing (Figure 3). The manufacturing can be performed at a very low cost by means of multi-jet fusion (MJF) or selective laser sintering (SLS) of PA12, an engineering-grade nylon 12 powder with effective electrical insulating properties, mechanically durable and chemically resistant. The only drawback of PA12 is its relatively low glass transition temperature of 55 °C, thus a different material should be used if significantly higher environment temperatures are expected. The SMU enclosure features a transparent Plexiglas window that allows the visualization of the status LEDs: the power led turns red when the board is correctly powered and green when a successful communication is established with the driver; the data led turns yellow when the SMU begins the data acquisition; the link led blinks blue when the PPS signal is received.

### 2.2. Software

**Figure 4:**
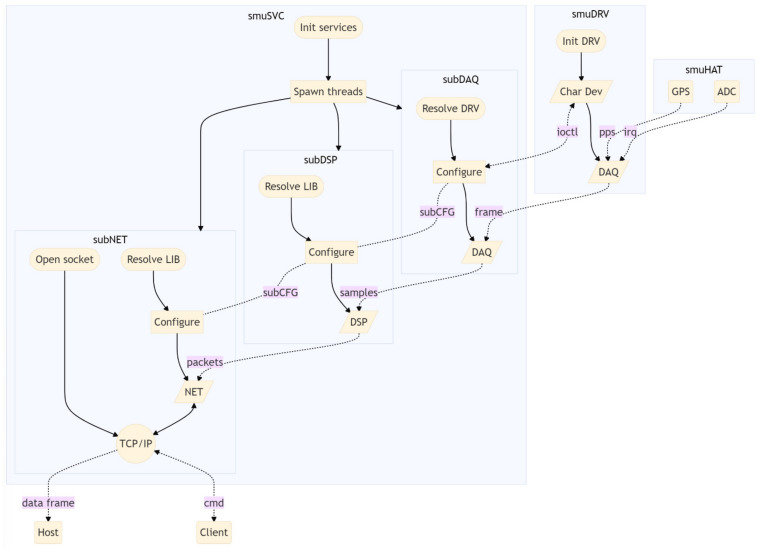
Software architecture: the driver provides access to the hardware resources via standard IOCTL system calls; the main service is composed of 3 sub-modules that run on separate threads.

The software layer (Figure 4) includes several blocks: a driver (smuDRV) to interface the hardware module; a data acquisition service (smuSVC) that runs in background; a remote configuration tool (smuGUI); a shared library (smuLIB) with common code and data types; two plugins in the form of dynamic libraries that can be swapped at run-time, one for data processing (smuDSP) and one for network and communication purposes (smuNET).

#### 2.2.1. smuDRV

The SMU driver is a kernel module [24] that allows to interface the hardware at the lowest admissible level in the operating system. It configures all the required hardware peripherals of the microprocessor by direct registry manipulation and maps the memory between kernel space and user space.

As mentioned above, the low latency synchronization performance and high data rate of the SMU are achieved with a combination of SMI interface and DMA techniques. The reason for this solution stems from a big limitation of the RPi’s CPU: the ARM architecture of the Broadcom BCM2837 SoC does not support the configuration of an interrupt vector table (IVT) when used in conjunction with an operating system, but only in bare-metal mode. This means that it is not possible to register the memory address of a specific interrupt request (IRQ) handler function to be called upon hardware interrupt and therefore interrupts must be handled by the operating system, spawning a thread that polls the IRQ register. This mechanism is more than sufficient in most standard operations, providing in the user-space a typical latency of few hundred microseconds. Realtime (RT) operating systems generally offer better performance over non-RT systems; alternatively, kernel patches such as the Preempt-RT can be used to improve the system response. Unfortunately, even in this case, the achievable latencies are in the order of tens of microseconds, still orders of magnitude higher than those required for industrial applications. When the interrupt is handled directly within the kernel-space, the latency can be further reduced to a minimum of 4 µs, which can increase up to few tens of microseconds depending on the CPU load [13,22]. To overcome this limitation, the SMU combines DMA and SMI to provide a synchronization latency in the order of few hundred nanoseconds. At the same time, this approach also provides a solution for high-speed data transfers paced by the ADC data ready interrupt. However, the synchronization and data ready signals have to be combined together since the SMI interface has only one hardware interrupt pin.

**Figure 5:**
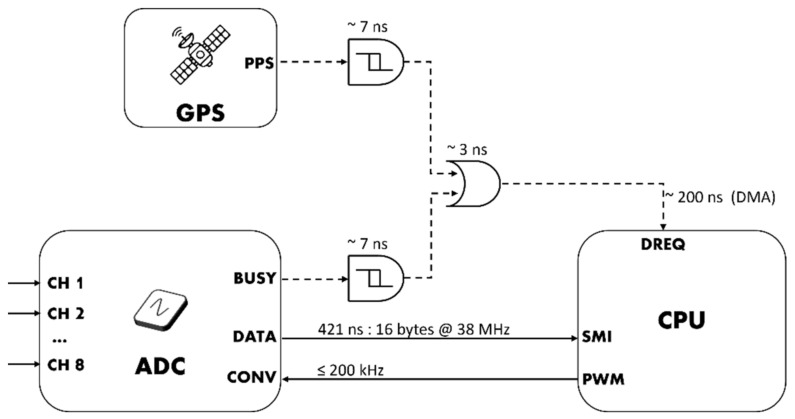
Interrupt conversion and detection scheme; propagation time is reported next to the respective logic gate, whereas for DREQ the time refers to DMA latency.

As shown in Figure 5, the PPS and ADC interrupts are first converted from edge- to level-sensitive interrupts and then combined with a logic OR in a single signal that is fed to the DREQ pin of the SMI. When the DREQ line is asserted low, the SMI paces the execution of a chain of DMA control blocks, which perform the required operations directly manipulating the hardware registers at machine level.

**Figure 6:**
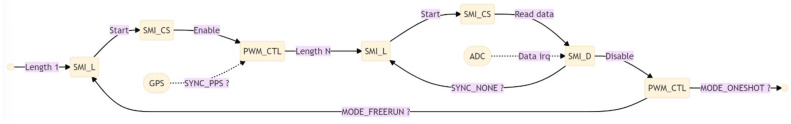
State diagram of the DMA finite state machine for synchronization and data acquisition.

**Figure 7:**
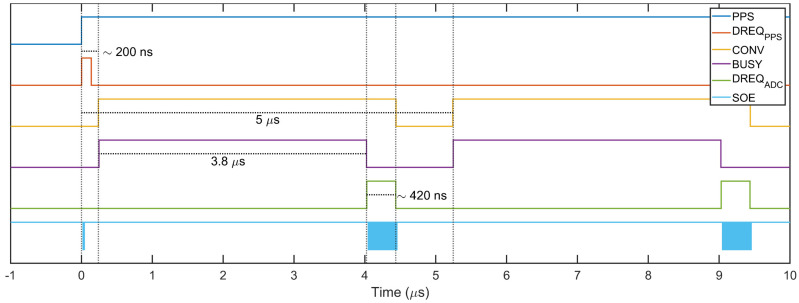
Timing sequence from logic analyzer for a sample rate of 200 kS/s.

The sequence (Figure 6) starts by configuring and starting the SMI for a transfer of length 1 (1 dword or 4 bytes) from the bus memory into the PWM Status register; this dword is composed by a proper combination of configuration bits, in which the PWM enable bit is set to 1. If the PPS synchronization is active, the DMA is halted and the transfer will take place upon DREQ interrupt. When the PPS interrupt occurs, the signal is converted in the short pulse DREQ_PPS_ in Figure 7 that releases the previously halted DMA sequence. As programmed, the DMA copies the dword in the PWM register triggering the immediate generation of the conversion pulses, after a typical delay of 200 ns. The DMA sequence then proceeds by configuring and starting the SMI for a transfer of bytes,

(1) $$N=N_{B}\cdot N_{ch}\cdot F_{s}\;$$

where N is total number of bytes paced by DREQ signal, $N_{B}=2$ is the number of bytes per channel, $N_{ch}=8$ is the number of channels and $F_{s}$ is the desired sample rate. This corresponds to acquiring 1 s of data from all the channels at the given sample rate.

When a conversion pulse is generated, the ADC successive approximation register begins the conversion and the BUSY line is pulled high until the process is completed, approximately after 3.8 µs. Upon end of conversion, the BUSY falling edge is converted to a DREQ_ADC_ pulse with duration

(2) $$t_{x}=\frac{N_{B}\cdot N_{ch}}{F_{SMI}}\;,$$

and the output enable line SOE toggles $N_{B}\cdot N_{ch}$ times at $F_{SMI}=38\;\mathrm{MHz}$ allowing to transfer the acquired samples from 8 channels (16 bytes) in approximately 420 ns.

As a result, the implemented mechanism is very similar to a hardware finite state machine (FSM), which is highly reliable since it is autonomous and therefore mostly independent and not influenced by the CPU load. At the same time, the data acquisition FSM leaves the CPU fully unused and the computational power of the 4 cores can be dedicated to data processing and auxiliary functions.

#### 2.2.2. smuSVC

**Figure 8:**
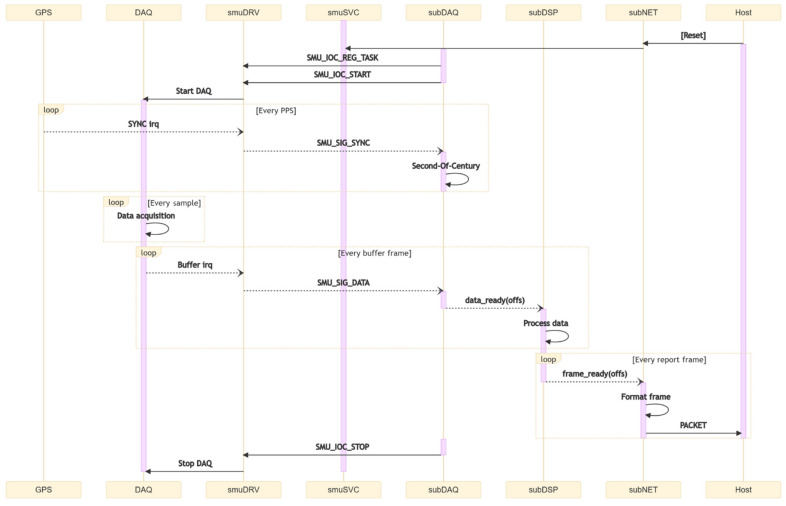
Sequence diagram of standard SMU operation. Arrows show the flow of information: system calls (solid) right-to-left and system signals (dashed) left-to-right.

The main service or daemon is a background routine that is executed in the user space [25]. It consists of three separate threads that perform different operations (Figure 4). The thread subDAQ configures the driver by defining the sample rate, synchronization mode and operating mode. It controls the status of the driver via standard IOCTL instructions and receives back signals to control the data flow (Figure 8). The other two threads, respectively subDSP and subNET, are wrappers for the execution of flexible data processing and networking functionalities. They expose three slots, *lib_allocate*, *lib_destroy*, and *lib_process*, that receive the dynamic binding at runtime of external dynamic libraries developed using the smuDSP and smuNET plug-ins.

To start the acquisition, the daemon first opens the driver, which in turns stores the task ID of the daemon, later used by the driver to forward signals back to the daemon. Subsequently, the daemon sends the start signal and the driver begins to listen for a synchronization interrupt; when this is received the driver enables the conversion base and sends back a synchronization signal that is used by the daemon to calculate the timestamp.

Samples acquired by the ADC are transferred first into the SMI buffer and then copied by the DMA into the allocated un-cached memory. This coherent memory, i.e., automatically synchronized with a virtual memory counterpart, is mapped by the driver into the user space, giving the daemon the possibility to direct access the data. However, the daemon still needs to know the exact position in the buffer where the most recent data are written, thus the driver periodically informs the daemon by emitting a signal that carries a memory position index as argument. To avoid overloading the operating system of signals, the driver accumulates data in the buffer for 10 ms before notifying the daemon, corresponding to a buffer frame rate of 100 Hz that can be changed if required.

#### 2.2.3. smuDSP & smuNET

The processing and network plugins are dynamic libraries [26,27] with three entry-point functions that are linked at runtime to the correspondent daemon slots in subDSP and subNET: *_init* and *_exit* perform the required memory allocation/deallocation, the initialization and exit steps, respectively; *_proc* is the core function for data processing, formatting, and logging. This architecture allows to easily replace the processing libraries at run-time, potentially supporting in a future release the parallel simultaneous execution of multiple plugins. Additionally, the separation between the core service and the plugins allows the development of libraries with different licensing scheme, able to seamlessly integrate with the platform.

**Figure 9:**
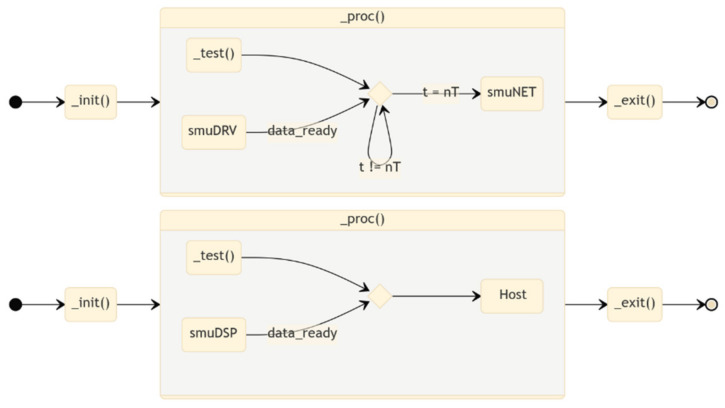
State diagram of the suds (**top**) and smuNET (**bottom**) plug-ins.

When a buffer frame is filled, the driver emits a *data_ready* signal that is received by the daemon and forwarded to the linked smuDSP plugin, together with the memory position with fresh data. The plugin will perform the required processing, timestamp the results and populate the output buffer. When the timestamp matches an integer multiple of the reporting period (Figure 9), the smuDSP plugin emits in turn a second *data_ready* signal that is received by the smuNET plugin. This will pack the data according to the desired data protocol and send them to a defined network host.

### 2.3. Test Environment

The repository provides a flexible testing environment for MATLAB, developed using the Data Acquisition Toolbox in conjunction with any DAQ board supported by the same toolbox. Requirements for the selection of the DAQ board include (i) the ability to generate/acquire analog and digital waveforms with the same clock, thus guaranteeing the absence of trigger skew, (ii) an analog output range of ±10 V, and (iii) a sampling rate of 1 MHz at least.

The repository includes a generic test suite for automatic measurements [28] and one for the use of the SMU as phasor measurement unit (PMU) [29] in power systems applications. The former provides support for standard static and dynamic tests, such as linear sweep of the inputs and frequency response, and can be used as a template for developing specific tests. The latter provides support for testing the compliance of the device with the international standard IEC/IEEE 60255-118-1 for synchrophasors in power systems [30]. It generates the required test signals and acquires the raw data from the SMU for further processing in MATLAB, allowing to compare different algorithms performance and finely tune the algorithms to compensate the group delay.

## 3. Results and Analysis

The experimental setup adopted for the SMU characterization mainly consists of a NI USB-6356 data acquisition board and by National Instruments, a high-performance DAQ device with synchronous mixed-signals IO capabilities up to 1 MS/s. This is used to generate the analog test signal with an estimated worst-case accuracy of 331 ppm over the entire full scale ±10 V, under the assumption of 1 °C variation from the last auto-calibration and 20 °C from the last external calibration [31]. As the analog and digital sub-blocks of the DAQ share the same 100 MHz clock, it is possible to generate perfectly synchronous mixed-signal waveforms, with 10 ns time base resolution and no inter-channel delay thanks to absence of any DAC reconstruction filter. The timing accuracy declared by the manufacturer is 50 ppm of the sample rate. Additionally, a 16 channels logic analyzer Logic Pro 16 by Saleae was used for timing measurements, with a sample rate of 500 MS/s that allows to achieve a time resolution of 2 ns and an oscillator accuracy of 50 ppm.

Characterization tests were performed to assess the SMU performance under static, dynamic, and transient conditions. Testing procedures are described in detail in the following section together with the results, which are summarized in Table 1. While ideal performances are limited by the specifications of the ADC, actual ones also depend on the additional components of the analog front-end and on the overall system design.

Additionally, performance results for the possible use of the SMU as phasor measurement unit (PMU) are also reported. However, in this case, collected measurements are not exclusively representative of the SMU performance, but also depends on the specific processing algorithm for phasor estimation and its implementation.

### 3.1. Static Tests

The static characterization is carried out to quantify the accuracy of the system, by applying a linear sweep to the input $v_{i}$ over the entire full-scale range (FSR), while measuring the digital output of the ADC. The corresponding measured output voltage is then calculated from the ADC readings as

(3) $$v_{o}={\mathrm{ADC}}_{LSB}\cdot \frac{\mathrm{FSR}}{2^{n}},$$

where n=16 is the number of bits. The SMU static transfer characteristic can be described by a linear relation with voltage gain G and offset $V_{os}$

(4) $$v_{o}=G\cdot v_{i}+V_{os},$$

which includes the effects of the actual LSB size, AFE loading, signal attenuation introduced by the filters and ground mismatches. Conversely, non-linear effects such as quantization error and integral nonlinearity are not accounted by (4), but rather included in the dynamic characterization in the form of noise and harmonic contribution, respectively.

**Figure 10:**
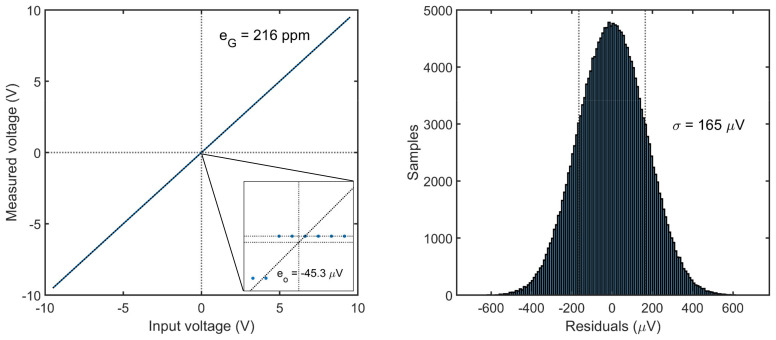
Systematic errors of the static characteristic (**left**) and linear regression residuals (**right**).

The parameters in Equation (4) are estimated via linear regression of the acquired N samples, by means of ordinary least squares (OLS) method. Given the parameters vector β and the regressors matrix X, the method requires to solve the linear problem

(5) $$v_{o}=X\beta ,\;\mathrm{with}\;\beta =\left[\begin{array}{c} V_{os}\\ G \end{array}\right],\;X=\left[\begin{array}{cc} 1 & v_{i}\left(0\right)\\ 1 & v_{i}\left(1\right)\\ \vdots & \vdots \\ 1 & v_{i}\left(N\right) \end{array}\right]$$

to derive the best estimation of the parameters $\hat{\beta }$. Hence, the offset error and gain error can be expressed as

(6) $$e_{O}={\hat{V}}_{os},\;\;\;e_{G}=\hat{G}-1,$$

and respectively quantified as −45.3 µV and 216 ppm (Figure 10), which is far below the accuracy declared by the manufacturer of ±150 µV and ±1220 ppm [32]. The uncertainty associated with this estimation can be evaluated from the covariance matrix of the residuals under the assumption of errors homoscedasticity (i.e., constant variance of the residuals), but it was found to be much smaller than the manufacturing tolerance [21].

### 3.2. Dynamic Tests

The dynamic characterization aims at assessing the frequency response of the SMU, as well as its performance at 50 Hz for power grid monitoring applications, evaluating their degradation due the additional system noise as well the non-ideal sampling clock.

**Figure 11:**
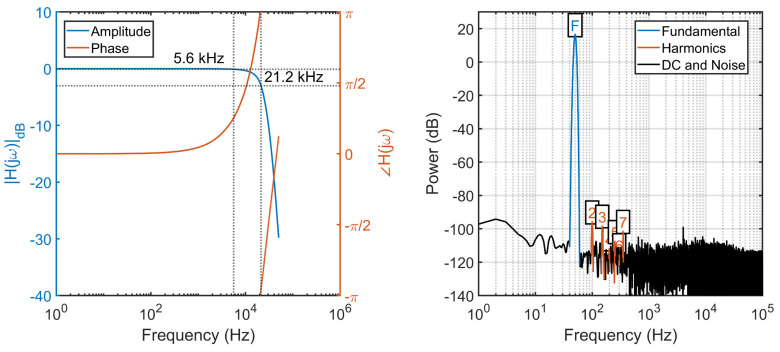
Frequency response (**left**) and power spectrum (**right**) at 50 Hz.

The frequency response is obtained by sweeping the frequency of a sinusoidal input signal with amplitude ±9.5 V and duration T = 1 s in the range [0, $F_{S}/2$], where $F_{S}$ is equal to the maximum SMU sample rate of 200 kS/s. For each acquired signal, the discrete Fourier transform (DFT) is calculated, and results are presented in Figure 11. The 21.2 kHz signal bandwidth (−3 dB), with a measured flatness (−0.1 dB) of 5.6 kHz, is sensibly smaller than the one reported in the ADC datasheet, but this is due to the additional low-pass input filters in the analog front-end, which can however be removed if not required. In most applications these bandwidth values are more than sufficient, including for instance audio applications with signal bandwidth up to 22 kHz, low frequency earthquake monitoring, but also power grid monitoring where an accurate power flow calculation typically requires monitoring up to the 25th of harmonic of the line frequency. Furthermore, the flatness of the SMU frequency response easily supports measurements above the 35th harmonic as requested by the standard IEEE 519 for Harmonic Control in Electric Power Systems, with a class of accuracy as low as 0.1%.

For evaluating the performance of the SMU in power grid monitoring applications, a 50 Hz test signal is acquired to estimate the signal-to-noise ratio (SNR) and the total harmonic distortion (THD). The estimated SNR of 92.1 dB and THD of −109.4 dB are largely consistent with those declared by the manufacturer, 92.7 dB and 114 dB, respectively, demonstrating a negligible degradation of the performance with respect to those of the bare ADC tested by the manufacturer in an ideal environment. This is particularly important for harmonic estimation in Power Systems, where the achieved THD would allow to perform distortion measurement with an accuracy of 3.8 ppm over the entire signal bandwidth. The achieved performances are more in general quite remarkable, especially from the noise point of view, considering that the SMU is an integrated low-cost mixed-signal system where high-speed digital lines run in close proximity to analog lines and where the power supply lines are shared by both the CPU and the ADC.

### 3.3. Transient Tests

**Figure 12:**
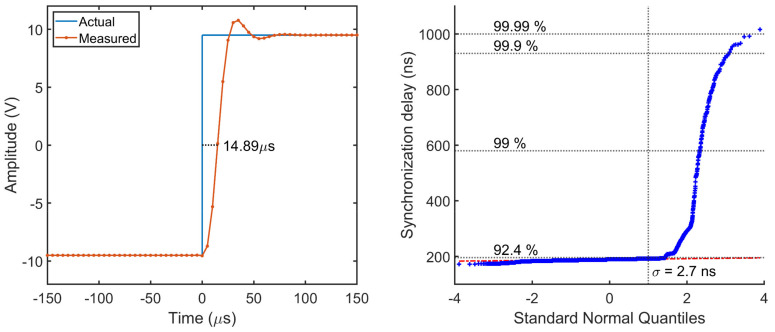
Step response (**left**) and synchronization delay quantile-quantile plot (**right**).

The transient characterization is performed to assess both the group delay and the synchronization delay of the SMU. The former is evaluated from the step response, when the input is instantaneously varied across the entire full-scale range and is measured as the time required by the output to reach the 50% of the final steady state value. Results in Figure 12 show a group delay of 14.9 µs, with a 13.4% overshoot and a settling time of 63 µs. This delay is quite consistent with the typical 13 µs value declared by the manufacturer, whereas the difference is only in minimal part due to the synchronization delay.

The delay introduced by the SMU in the synchronization with the time reference signal was evaluated from N = 10^4^ measurements. Results in Figure 12 show an average delay of 188.7 ± 2.7 ns in the 92.4% of the cases, which is between 2 and 3 orders of magnitude smaller than best performance achievable on a real-time OS with kernel preemption. In a small percentage of cases, between 92.4% and 99%, the delay can reach up to 580 ns, and 930 ns in 0.9% of the cases. Exceptionally, only in one case the synchronization delay has reached 1 µs.

While these unprecedented results are clearly due to the extremely low latency of the SMI interface and independence from the OS activity, still it can be observed a non-deterministic nature of the delay that can only be ascribed to the DMA. In fact, the system bus used to exchange AXI bus request is shared by all peripherals and therefore the maximum bandwidth that a DMA channel can consume is limited. When the system bus bandwidth is mostly free, the DMA can load and execute the control block responsible for generating the time base with the lowest admissible latency, resulting in a synchronization delay of 172 ns. As the DMA requires a fixed number of clock cycles to execute a control block, this latency value depends only on the internal clock frequency. Conversely, in presence of other system requests the internal arbitration system can temporarily delay a DMA request to accommodate for other bus requests, thus increasing the synchronization delay as observed in Figure 12.

### 3.4. Phasor Estimation

When the SMU is used as PMU, performance must comply with the relative international standard IEC/IEEE 60255-118-1:2018 [30], which disciplines the use of synchrophasors for power systems. The standard defines synchronized phasor, frequency, and rate of change of frequency (ROCOF) measurements, including their respective errors: total vector error (TVE), frequency error (FE) and rate of change of frequency error (RFE). Additionally, the standard defines time tag, synchronization requirements and methods for evaluating the PMU compliance under static and dynamic conditions.

Conversely, the standard does not define hardware, software and methods potentially required to satisfy the stated requirements. This makes the achievable performance strongly dependent on a specific combination of these factors and very sensitive, for the same hardware solution, to different processing algorithms. Furthermore, the standard defines two classes of accuracy, one for power grid measurements (M) and one for protection (P) purposes, each best covered by specific algorithms.

For this reason, since the SMU is an open-source data acquisition platform that can also be used for several applications including, but not restricted to, phasor measurements, results for PMU application are reported in relation to a specific algorithm included in the repository, the interpolated modulated sliding discrete Fourier transform (Ip-mSDFT). This algorithm is characterized at the same time by high accuracy, high throughputs, and low-computational complexity, allowing to achieve reporting rates in the order of thousands of phasors per second [33,34].

**Figure 13:**
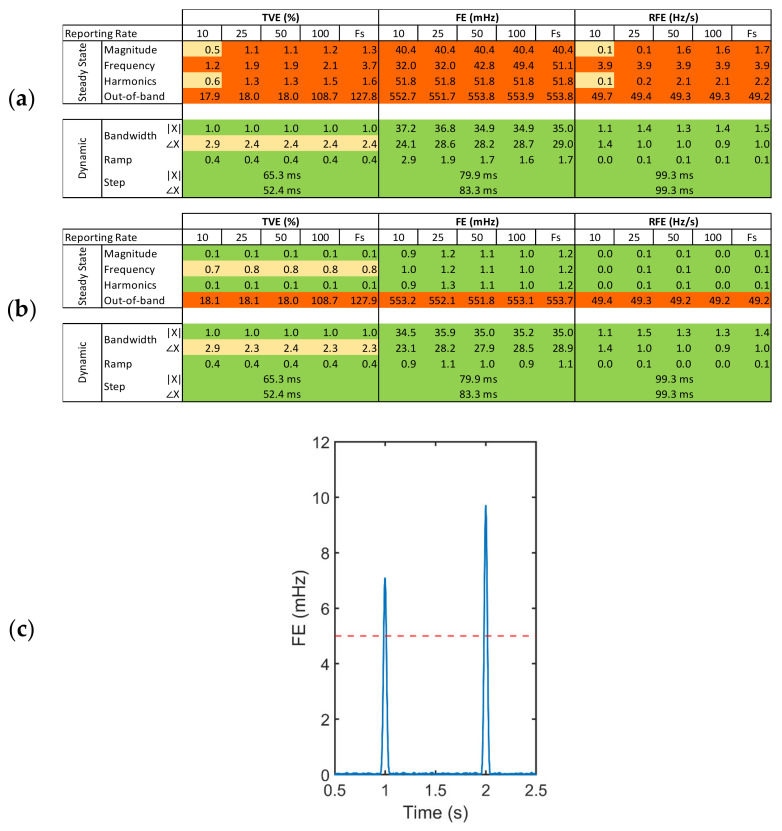
Compliance test evaluated over the full interval (**a**) and excluding the synchronization instants (**b**). Results are divided in categories: very good are below 30% of the limit imposed by the standard (green), acceptable are above 30% but below the limit (yellow), failed are above the limit (red). Frequency error (**c**) at 53.4 Hz and standard compliance limit of 5 mHz in red.

Figure 13 shows that excluding the synchronization instants (c) that lead to the results in (a), the overall performance of the device is largely compliant (b) with the standard. The errors exceed the thresholds in correspondence of every PPS due to the realignment of the drifting time base with respect to the external reference signal. Since the algorithm performs an estimation of the phasor over a sliding time window of 60 ms (3 cycles at 50 Hz), the discontinuity induces an increase of the error that vanishes within the duration of such window (c). A very simple way of avoiding this issue is to interpolate the signal in real-time during the acquisition before proceeding with the phasor estimation. Regarding the non-compliance of the out-of-band type of signal, this is a well-known issue for many synchrophasor algorithms, including the tested iDFT. More complex algorithms based on the Taylor Fourier transform may be adopted to achieve better performance with this type of signals [35]. In addition, it is worth noting that the compliance to the out-of-band interference limits is not required if a reporting rate lower than 10 measurements per second is used.

## 4. Conclusions

The presented results demonstrate that the performance of the SMU as a generic data acquisition board mainly reflect the performance of the adopted ADC. The overall accuracy of the static transfer characteristic is far below 1 LSB for both the offset and gain error. Although clock non-idealities (jitter and intermodulation) typically degrade the SNR increasing the noise floor, dynamic tests have shown that the time base generated by the internal PLL of the Raspberry Pi has negligible impact. Furthermore, the additional ability of the SMU to synchronize its time base to an external trigger event, generally a GPS reference, introduces a typical delay in the sub-microsecond range and therefore negligible in most of the applications. Tests for the use of the device as PMU for power grid monitoring applications have also shown that, if proper pre-processing algorithms are implemented, the overall performance of the SMU is largely compliant with the standard.

These results are relevant since they overturn the common belief that embedded devices running on an operating system, such as single board computers, are not suitable for high performance direct data acquisition. This belief is grounded on the lack of hardware interrupt support within an operating system environment and on the unpredictable nature of the job scheduler, which introduces indeterministic delays in timed operations, leading to loss of samples, or even system stall.

The work carried out by the authors provides for the first time an open-source platform for implementing high-performance data acquisition systems on low-cost single-board computers. The SMU can be used as a standard DAQ device, or with enhanced synchronization capabilities, unlocking an effective access to the implementation of all those systems that require, for instance, geographically synchronized distributed measurements. Furthermore, researchers and engineers working on a specific application would be able to easily execute their high-level code (e.g., python) on board, without having to design specific firmware for DSP/FPGA, thus benefiting from the layers of abstraction and flexibility provided by an operating system.

## Figures and Tables

**Table 1:** SMU performance characterization summary.

Test	Symbol	Description	Value	Unit
Static	$e_{O}$	Offset error	−45.3	µV
	$e_{G}$	Gain error	216	ppm
Dynamic	${\mathrm{BW}}_{3\mathrm{dB}}\;$	Signal bandwidth	21.2	kHz
	${\mathrm{BW}}_{0.1\mathrm{dB}}$	Flatness	5.6	kHz
	SNR	Signal-to-Noise Ratio	92.1	dB
	THD	Total harmonic distortion	−109.4	dB
Transient	$t_{d}$	Group delay	14.9	µs
	$t_{s}$	Settling time	63	µs
	PO	Percentage overshoot	13.4	%
	$t_{x}$	Synchronization delay	188.7	ns

## Data Availability

Design files, codes and documentation of the SMU are available open-source in the original public repository: https://git.rwth-aachen.de/acs/public/automation/smu (accessed on 3 July 2022).
